# Mutations associated with resistance to rifampicin and isoniazid identified in strains of the *Mycobacterium tuberculosis* complex by GenoType MTBDRplus in Panama, 2015–2021

**DOI:** 10.1128/spectrum.02400-24

**Published:** 2025-06-09

**Authors:** Juan Domínguez González, Juan Castillo Mewa, Prudencio González, Pedro Del Cid, Jacinto Ariel Pérez Ruíz, Samantha Eunice Rosas Hermosilla

**Affiliations:** 1 Instituto Conmemorativo Gorgas de Estudios de la Salud 199319, Panama City, Panama; ICON plc, Health Economics and Epidemiology, London, United Kingdom

**Keywords:** tuberculosis, mutation, *Mycobacterium tuberculosis*, genotype, drug resistance, Panama

## Abstract

Tuberculosis is one of the diseases causing high rates of morbidity and mortality in several countries. However, efforts in the use of diagnostic methods for tuberculosis and the detection of drug resistance are essential to reduce cases. Since 2015, rapid molecular diagnostic tests have been implemented in Panama, enabling the detection of drug resistance, mainly rifampicin and isoniazid, in patients with suspected tuberculosis. This study aimed to identify mutations in *Mycobacterium tuberculosis* complex strains with resistance to rifampicin and isoniazid using GenoType MTBDRplus. It is a retrospective study reviewing the results of the GenoType MTBDRplus version 2.0 test from 2015 to 2021. Strains not identified as *Mycobacterium tuberculosis* complex and those that did not show a mutation pattern and were categorized as sensitive were excluded. Data analysis was carried out using the Chi-square tests, Pearson’s Correlation, and principal components analysis. A total of 4,301 strains were analyzed, of which 8.8% were detected with mutation or resistance probes in one or more of the genes analyzed: 56.0% in the *rpoβ* gene, 11.9% in the *inhA* gene, and 8.2% in the *katG* gene. In addition, other mutations such as *rpoβ/inhA* and *rpoβ/katG* were detected in 9.5% and 13.5% of cases, respectively. Thirty-eight resistance patterns were identified, with H526D and S531L being the most frequent mutations in the *rpoB* gene, and S315T1 and C-15T are the most common in *katG* and *inhA*, respectively. The resistance patterns detected by the GenoType MTBDRplus assay highlight the genetic variability of drug resistance in the country and emphasize the need to implement epidemiological surveillance methodologies. Integrating patient clinical data with genetic variation information is essential for improving disease control and understanding transmission dynamics and drug resistance acquisition. These findings also provide important insights for guiding tuberculosis treatment strategies in Panama, supporting the use of molecular tools for the early detection of drug resistance, enhancing our understanding of the epidemiology, and informing clinical decision-making.

**IMPORTANCE:**

This study focuses on understanding how *Mycobacterium tuberculosis* strains in Panama develop resistance. With tuberculosis (TB) cases becoming harder to treat due to drug resistance, especially after the disruptions caused by the COVID-19 pandemic, rapid and accurate diagnosis is crucial. By using advanced molecular tests to identify specific genetic mutations in drug-resistant TB strains, this research helps improve treatment decisions, leading to better outcomes for patients. Understanding these mutations also aids in controlling the spread of TB. Given the rising global concern over drug-resistant TB, the findings of this study are important not only for Panama but also for other regions facing similar challenges.

## INTRODUCTION

In 2022, there was a 3.1% increase in the incidence of tuberculosis cases with multidrug resistance (MDR-TB) or resistance to rifampicin (RR-TB) compared to 2020. It was estimated that 3.6% of new cases and 18% of previously treated cases were affected by RR-TB/MDR-TB (1). This situation has been attributed to the impact of the COVID-19 pandemic on tuberculosis case detection. Tuberculosis (TB), caused by the *Mycobacterium tuberculosis* complex (MTBC), is a preventable and curable infectious disease that contributes to significant morbidity and mortality rates in various countries (2). Nevertheless, efforts in implementing early and accurate diagnostic methods for all forms of tuberculosis, as well as rapid detection of drug resistance, are fundamental in reducing the number of cases (3).

When clinical suspicion of TB arises, the initial step involves microbiological confirmation of the bacillus, employing the most recent and recommended rapid molecular diagnostic tests. These methodologies possess the potential to promptly diagnose TB and provide precise results regarding mutations associated with anti-tuberculosis drug resistance, which aids in selecting an effective treatment approach (4). The Pan American Health Organization and the World Health Organization (WHO) consistently issue updates on the use of rapid molecular tests as an initial diagnostic tool for TB and drug resistance, including line probe assays for detecting MTBC and resistance to first and second-line tuberculosis treatment drugs (3, 5, 6). Resistance is identified through mutations in chromosomal genes of actively growing bacteria, as well as epigenetic modifications in gene expression and protein alterations that confer drug tolerance in bacteria. Both types of resistance pose significant challenges in effectively controlling the disease (7).

Estimations for 2021 revealed an incidence of 1,800 TB cases (uncertainty range of 1,400–2,300) in Panama, with a rate of 42% per 100,000 population. Among these cases, approximately 4.2% of new cases and 12% of previously treated cases were estimated to have RR-TB/MDR-TB, as indicated by the country profile generated by the WHO in 2023.

Since 2015, rapid molecular diagnostic methodologies have been implemented in Panama to determine drug resistance in patients suspected of having TB, primarily targeting rifampicin (RIF) and isoniazid (INH). Although data on patients resistant to these drugs are available, there has been no specific analysis conducted on the mutations associated with drug resistance, which could contribute to enhanced clinical management of patients with drug-resistant tuberculosis.

To address this gap, our study is the first to analyze these mutations using a larger data set within this specific population, covering the period from 2015 to 2021. This broader scope allowed the identification of new trends in mutation frequencies and resistance patterns that had not been previously documented, offering updated insights into the current epidemiology of drug-resistant tuberculosis in the country.

The objective of this study was to identify mutations in strains of the MTBC exhibiting resistance to RIF and INH by employing the GenoType MTBDRplus assay.

## MATERIALS AND METHODS

### Study population and period

This is a retrospective study that involved reviewing the results of the GenoType MTBDRplus version 2.0 test to determine mutations associated with RIF and INH resistance in strains of MTBC. The study was conducted at the Mycobacteriology Section of the Gorgas Memorial Institute of Health Studies between 2015 and 2021. Strains that were not identified as MTBC, strains of MTBC without detected mutations or with interpretations that did not indicate possible mutations, and strains categorized as drug-sensitive were excluded from the analysis.

### Molecular hybridization procedure

Strains isolated from solid culture media such as Lowenstein Jensen or BD MGIT 7 mL were subjected to bacterial DNA extraction using the Genolyse kit protocol. Amplification and hybridization were performed using the GenoType MTBDRplus version 2.0 kit. Amplification was conducted on the GeneAmp PCR Systems 9700 Applied Biosystems instrument, and hybridization was performed in the Twincubator (8–11). As negative controls, molecular-grade water and the ATCC 25177 strain of drug-sensitive *Mycobacterium tuberculosis* complex were included, with both controls being included from the extraction process to the hybridization step (12). Additionally, this assay has been included in the quality control program sent by the Supranational Tuberculosis Laboratory to the National Reference Laboratories, yielding satisfactory results.

### Data collection and statistical analysis

The collected data were tabulated in Microsoft Excel tables, where the frequency and percentage of each detected band, according to the involved genes and their possible mutations, were determined. Data analysis was performed using chi-square tests, Pearson’s correlation, and principal component analysis. The significance level was set at *P ≤ 0.05 and **P ≤ 0.01. The JMP 14.0 software and the RStudio platform with R version 4.2.1, along with the dplyr, MASS, tidyr, and FactoMiner packages, were used for the data analysis.

## RESULTS

### Mutations associated with rifampicin and isoniazid in strains of the *Mycobacterium tuberculosis* complex from 2015 to 2021

A total of 4,301 strains of the *Mycobacterium tuberculosis* complex were analyzed using GenoType MTBDRplus version 2.0. An 8.8% (377/4,301) of strains with mutation probes or conditions suggesting resistance in one or more of the analyzed genes were detected, as shown in Table 1: 56.0% in the *rpoβ* gene, 11.9% in the *inhA* gene, and 8.2% in the *katG* gene. Furthermore, mutation probes were detected in *rpoβ*/*inhA* and *rpoβ*/*katG* in 9.5% (36/377) and 13.5% (51/377) of cases, respectively. Only one strain (0.3%, 1/377) exhibited mutation probes in the *inhA/katG* genes, and two strains (0.5%, 2/377) showed mutation probes in all three genes analyzed in this study.

**TABLE 1:** Frequency of genes with mutation associated with resistance in strains of the *Mycobacterium tuberculosis* complex in Panama from 2015 to 2021

Year	Total strains analyzed	Total strains with mutation	*rpoβ*	*inhA*	*katG*	*katG*/ *inhA*	*rpoβ/ inhA*	*rpoβ/ katG*	*rpoβ/ katG/ inhA*
Total	4,301	377 (8.8%)	211 (56.0%)	45 (11.9%)	31 (8.2%)	1 (0.3%)	36 (9.5%)	51 (13.5%)	2 (0.5%)
2015	337	51 (15.1%)	21	6	5	1	7	10	1
2016	557	71 (12.8%)	35	8	6	0	11	11	0
2017	750	65 (8.7%)	36	5	6	0	9	9	0
2018	830	69 (8.3%)	40	8	5	0	2	13	1
2019	731	57 (7.8%)	40	5	5	0	4	3	0
2020	532	30 (5.6%)	16	7	2	0	3	2	0
2021	564	34 (6.0%)	23	6	2	0	0	3	0

**Fig 1:**
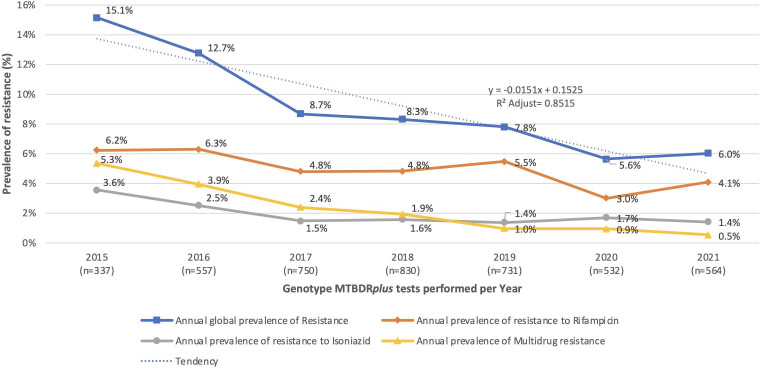
Prevalence of resistance according to genes with mutation associated with resistance in strains of the *Mycobacterium tuberculosis* complex in Panama from 2015 to 2021.

During the study period, the number of strains with mutation probes or conditions suggesting mutations in the *rpoβ* gene was higher than in the *inhA* and *katG* genes, as well as the conditions typically considered as MDRTB. Figure 1 displays the annual prevalence of resistance to RIF and INH, where the number of strains exhibiting resistance varied between 30 and 71 per year.

**Fig 2:**
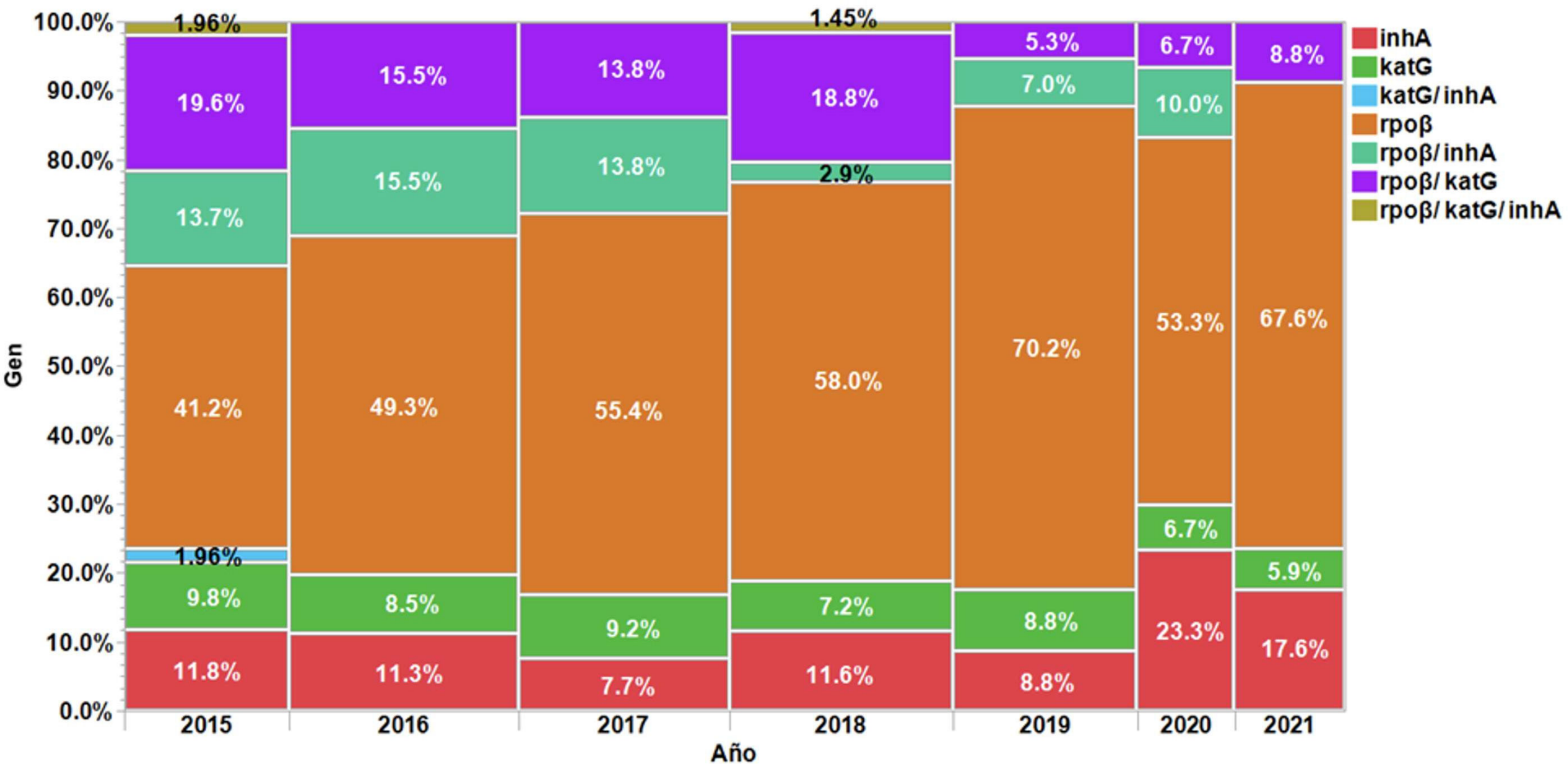
Distribution by year of genes involved in resistance in *Mycobacterium tuberculosis* complex strains. This figure presents the results of the proportions of reported resistance mutations from 2015 to 2021, where it can be observed that the proportion of appearance for each mutation per year remains constant (χ^2^ = 41.86, df = 36, and *P* = 0.23).

However, all prevalences of antibiotic resistance analyzed tend to decrease significantly over time (*P* < 0.05). Rifampicin resistance decreases at a rate of 0.44% per year (ANOVA *F* = 9.04, *P* = 0.03), while INH resistance decreases at a rate of 0.30% per year (*F* = 7.90, *P* = 0.04), and MDRTB decreases at a rate of 0.078% per year (*F* = 51.70, *P* = 0.001). Overall, the prevalence of resistance in this study is associated with time by 85.15% at a rate of 1.51% per year (*F* = 35.40, *P* = 0.0019). Meanwhile, Fig. 2 shows the annual proportion of each mutation, highlighting the dynamics of resistance over time.

### Patterns detected in genes conferring resistance to rifampicin and isoniazid based on the GenoType MTBDRplus assay

**Fig 3:**
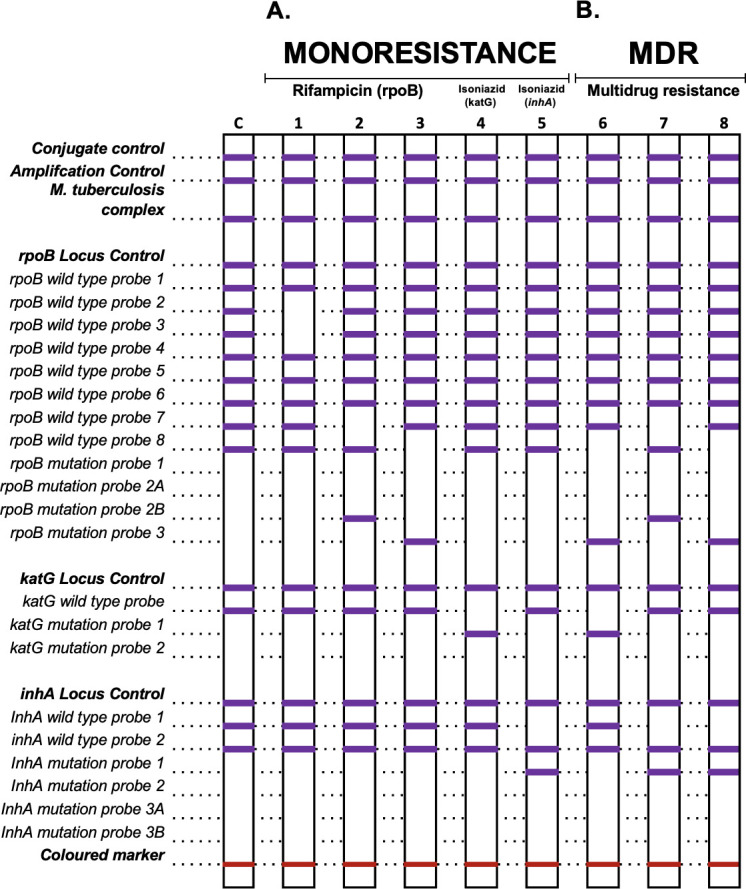
Representation of the most frequent patterns detected using the GenoType MTBDRplus assay. A: Monoresistance; B: MDR; C: control; 1, 2, and 3: mutation in *rpoβ* gene; 4 and 5: mutation in *katG* and *inhA* genes; 6: mutation in *rpoβ/ katG*; and 7 and 8: mutation in *rpoβ/ inhA*.

Following the assay guidelines, a total of 38 patterns were identified involving one or more of these resistance-conferring genes, respectively. The patterns with a frequency above 3.0% are shown in Table 2, where the mutation H526D in the *rpoβ* gene was identified with a frequency of 30.2%, corresponding to codons 526–529 (WT7), followed by the mutation S531L in codons 530–533 (WT8) with a frequency of 8.8%, and the inferred mutation in codons 510–517 (WT2/3) with a frequency of 3.4%. In the *katG* gene, the mutation S315T1 in codon 315 was identified in 6.4% of cases, and in the *inhA* gene, the nucleic acid mutation C-15T at position -15-16 (WT1) was found in 11.1% of cases. Figure 3 provides a visual representation of the most frequent patterns detected using the GenoType MTBDRplus assay, offering insight into their distribution and prevalence.

**TABLE 2:** Most frequent patterns in genes associated with resistance in *Mycobacterium tuberculosis* complex strains in Panama from 2015 to 2021

WT probe^*a*^	Codon/nucleic acid^*b*^	MUT probe^*a*^	Mutation detected	WT probe^*a*^	Codon/ nucleic acid^*b*^	MUT probe^*a*^	Mutation detected	Total	%
Gen rpoβ *n* = 211 (56.0%)									
ΔWT2/3	510–517		Unknown^*c*^					13	3.4
ΔWT7	526–529	MUT2B	H526D					114	30.2
ΔWT8	530–533	MUT3	S531L					33	8.8
Gen katG *n* = 31 (8.2%)									
ΔWT	315	MUT1	S315T1					24	6.4
Gen inhA *n* = 45 (11.9%)									
ΔWT1	-15-16	MUT1	C-15T					42	11.1
Gen rpoβ/ katG *n* = 51 (13.5%)									
ΔWT8	530–533	MUT3	S531L	ΔWT	315	MUT1	S315T1	44	11.7
Gen rpoβ/ inhA *n* = 36 (9.5%)									
ΔWT7	526–529	MUT2B	H526D	ΔWT1	-15-16	MUT1	C-15T	17	4.5
ΔWT8	530–533	MUT3	S531L	ΔWT1	-15-16	MUT1	C-15T	12	3.2

^*a*^ Wild-type band/mutation band identified within the GenoType MTBDRplus assay.

^*b*^ Position of the analyzed codon/nucleic acid.

^*c*^ Unknown: mutation not recorded by the assay.

In the detection of two or more mutations in the strain, the mutation S531L at codons 530–533 of the *rpoβ* gene, together with the mutation S315T1 at codon 315 of the *katG* gene, was detected in 11.7% of cases. Additionally, the mutation H526D at codons 526–529 of the *rpoβ* gene, along with the mutation C-15T at position -15-16 of the *inhA* gene, was identified in 4.5% of cases. Furthermore, the mutation S531L at codons 530–533 of the *rpoβ* gene, along with the mutation C-15T at position -15-16, was detected in 3.2% of cases.

Mutation H526D in the *rpoβ* gene showed a high frequency (136/300), followed by mutation S531L (100/300), and the remaining two mutations, H526Y (5/300)/D516V (4/300), were of low frequency, as shown in Table 3. Additionally, 55 isolates were registered with the absence of wild-type (WT) bands in the *rpoβ* gene, in which no mutant (MUT) bands were expressed (Table 3).

**TABLE 3:** Mutations in rifampicin and isoniazid according to drug resistance profile in *Mycobacterium tuberculosis* strains from 2015 to 2021^*a*^

	Total	MDRTB	RRTB	rHTB	*P*
Mutation in RIF					
Total	300	89	211		
*rpoβ H526D*	136	18	118		2.11 × 10^−14*b*^
*rpoβ S531L*	100	63	37		
*rpoβ H526Y*	5	0	5		
*rpoβ D516V*	4	2	2		
*rpoβ MUT not detected^c^*	55	6	49		
Mutation in INH					
Total	169	91		78	
*inhA C-15T*	82	38		44	0.0093^*b*^
*katG S315T1*	75	51		24	
*katG S315T2*	2	2		0	
*inhA T-8C*	1	0		1	
*katG not detected^c^*	8	0		8	
*inhA not detected^c^*	1	0		1	

^*a*^ MDRTB, multidrug-resistant, resistant to rifampicin and isoniazid; RRTB, resistant to rifampicin; and rHTB, resistant to isoniazid.

^*b*^ *P*-value by Chi-square test.

^*c*^ For the *P*-value by Chi-square test, undetected mutations in the genes were excluded.

Although this condition of absence of mutation bands occurs within the permissible codons defined by the GenoType MTBDRplus assay, the most frequent ones were codons 510–517 and 526–529, found in 14 and 13 strains, respectively. The S531L mutation exhibited high prevalence in isolates with mutations in the *katG* and *inhA* genes, while the H526D mutation was predominantly observed in isolates with single mutations in the *rpoβ* gene, as indicated in Table 2.

Among the 169 isolates with identified patterns in genes associated with isoniazid resistance, the *inhA* C-15T mutation accounted for 48.5% (82/169), *katG* S315T1 mutation for 44.4% (75/169), and 4.7% (8/169) showed the absence of the wild-type band, with associated mutations not detected. Mutations *katG* S315T2 and *inhA* T-8C were present in less than 2.0% of cases, as shown in Table 3. The *inhA* C-15T mutation was most detected among strains with mutations solely in the aforementioned INH-related genes, whereas the *katG* S315T1 mutation was more prevalent in strains with mutations in the *rpoβ* gene associated with rifampicin resistance.

**Fig 4:**
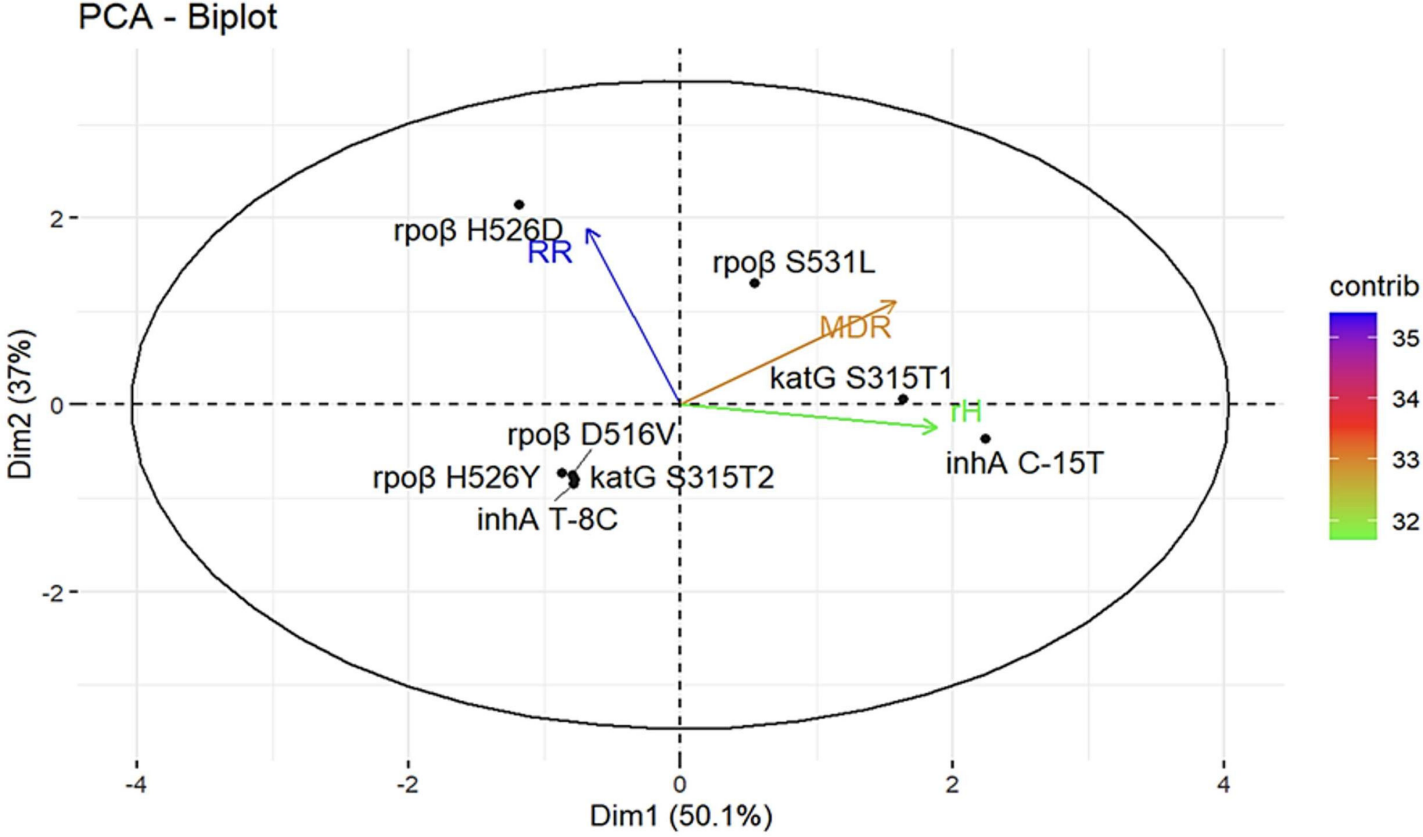
Comparative biplot depicting the association of rifampicin and isoniazid mutations according to drug resistance profiles in *Mycobacterium tuberculosis* strains from 2015 to 2021.

Figure 4 illustrates the genes most associated with drug resistance and their contribution to this relationship (indicated by the color gradient). The principal component analysis reveals that the *rpoβ* H526D mutation is more closely linked to RIF resistance, whereas *rpoβ* S531L is more associated with MDR strains. Additionally, *katG* S315T1 is also associated with MDR, but it is more specifically related to INH resistance, along with inhA C-15T. Hence, these genes collectively account for 50.1% of both MDR and INH resistance, while the *rpoβ* H526D mutation explains 37.0% of RIF resistance. In this study, these mutations together contribute to 87.1% of drug resistance overall.

The angles of the vectors suggest that MDR and *rpoβ* H526D are more likely to occur together. Consequently, RRTB falls within quadrant 4 because although it may occur in conjunction with MDR, it is relatively less frequently observed independently. Mutations in quadrant 3 indicate a strong association, meaning that the presence of one mutation is linked to the presence of the other, but they may not directly contribute to drug resistance. Being in quadrant 3 indicates interference rather than direct contribution, although such conclusions cannot be definitively established based on the available data.

These findings corroborate the results obtained through the Cochran-Mantel-Haenszel test, establishing an association between *rpoβ* mutations and MDR, RR, and time. Furthermore, they explain the correlation and dependence observed between time and changes in the frequency of resistance occurrences.

The Pearson’s chi-square test suggests differences in the frequency of mutations associated with MDR, RR, and rH resistance.

## DISCUSSION

The presented study is the second in the country focusing on mutations detected in strains with molecular-level resistance using a line probe assay (GenoType MTBDRplus), and it is the first one with larger data collected. This allowed us to identify a high frequency of strains with mutations solely associated with RIF resistance, despite mutations in INH being more common (13, 14). Although a higher number of mutations in the *inhA* and/or *katG* genes would be expected due to those detected in *rpoB* (considering that resistance to RIF is a marker of MDR-TB), the findings show a decrease in strains with mutations in both drugs (4, 15).

The decline in drug-resistant cases in our country can be attributed to the implementation of national strategies aimed at TB control and prevention. Key initiatives include the National Strategic Plan for TB Control (2023–2027) and Law 169 of 12 October 2020, which establishes the legal framework for TB prevention and management. Additionally, the National TB Control Program has strengthened TB control through the effective implementation of Directly Observed Treatment, Short-Course and updated treatment protocols in accordance with WHO recommendations. The decline in drug resistance also correlates with the integration of molecular testing into our diagnostic algorithms, enabling early detection and appropriate treatment, further contributing to improved TB management.

To better contextualize these local findings, it is useful to consider evidence from international studies that have explored mechanisms such as drug efflux pump induction (pharmacokinetic-pharmacodynamic variability) or silent mutations that facilitate the development of low-level, multi-drug mutations in *M. tuberculosis* strains (7, 15–18), which could be considered in this study due to the high number of resistance-associated mutations in RIF, encouraging subsequent research.

In this context, a deeper understanding of the specific genetic mechanisms driving resistance is essential. Unlike other pathogenic bacteria, resistance to anti-TB drugs in MTBC arises as a result of spontaneous chromosomal mutations in a specific gene called single-nucleotide polymorphisms that reduce the bacterium’s susceptibility to the antimicrobial agents and influence the efficacy of anti-TB treatments. Moreover, the primary mechanism of RIF resistance is due to mutations in the *rpoB* gene that encodes the DNA-dependent RNA polymerase β-subunit. RIF resistance is largely associated with the most common mutations of the *rpoB* gene within the 81 bp fragment of the RIF resistance-determining region or hot-spot region at codons *rpoB* 531, *rpoB* 526, and *rpoB* 516, between codons 507 and 533.

INH resistance appears more complex and has been associated with multiple genes, most commonly the *katG* gene that codes for a catalase-peroxidase and the promoter region of the *inhA* gene. INH resistance relies on the detection of mutations at codon 315 (*katG*315) and position -15 (*inhA*-15) in the promoter region.

The implementation of the GenoType MTBDRplus assay has optimized the rapid detection of mutations associated with RIF and/or INH resistance, facilitating the timely identification of MDR strains (2, 19). The results of this study confirm the presence of strains with exclusive molecular resistance to INH in the country. However, other mutations may exist in genes not covered by this assay, reinforcing the need to implement whole-genome sequencing of *Mycobacterium tuberculosis* as a complementary tool to improve diagnostic accuracy (18–20).

The patterns generated by the WT and/or MUT bands of the GenoType MTBDRplus assay provide valuable insights into the genetic variability of drug resistance in the country, which could help implement effective epidemiological surveillance methodologies.

Regarding the mutations detected in RIF, several studies have shown that mutations are more prevalent in the core region of the rifampicin resistance-determining region of the *rpoβ* gene (21). This study confirmed the localization of *rpoβ* gene mutations at codons 510–517, 526–529, and 530–533, which align with findings from other regional studies. Notably, approximately 70% of *rpoβ* gene mutations occur at codons 531 and 526, with H526D and S531L mutations being the most recurrent among strains with RIF resistance, even in MDR cases (22, 23).

Our study advances the understanding of drug-resistant TB in Panama by expanding both the temporal scope and the analytical depth of previous research. While the 2016 study provided a first evaluation of the GenoType MTBDRplus 2.0 assay applied directly to smear-positive respiratory specimens collected between 2012 and 2013 (mainly from pretreatment cases), our current study includes a broader and more recent data set from 2015 to 2021 (22). By analyzing a larger number of isolates and integrating resistance patterns with mutation profiles, we provide updated insights into the epidemiology of isoniazid and rifampicin resistance in the country. Furthermore, we identified key mutation combinations (e.g., *rpoB* S531L, *katG* S315T1, and *inhA* C-15T) that collectively explain 87.1% of drug resistance cases, offering valuable markers for surveillance and diagnostic strategies that were not characterized in earlier studies.

The *katG* S315T and *inhA*C-15T mutations of INH were more frequently identified in the group of resistant strains analyzed, in line with findings from other regional studies (2). It is worth mentioning that distinguishing between mutations when the pattern expresses resistance only to one drug and when it indicates resistance to both drugs is crucial. For example, the *rpoβ* H526D mutation is associated with single-drug resistance to RIF (30.2%), whereas the *rpoβ* S531L mutation is associated with only 8.8% of the cases. Additionally, MDR mutation patterns are associated with resistance primarily linked to the katG S315T gene of INH.

These findings differ slightly from those reported in other nearby countries, such as Colombia, where the predominant pattern was S531L (36.6%), while countries like Chile, Argentina, and Peru reported similar frequencies between 56% and 67% (24, 25). Mutation frequencies can vary across different geographical regions; for instance, the *rpoβ* 526 codon has been found in 10.5% in East Asia and 40% in China (26, 27).

The mutations occurring at different codons of the *rpoβ* gene might be associated with different levels of RIF resistance. Mutations at codons 526 and 531 are linked to higher levels of RIF resistance (minimum inhibitory concentration > 64 mg/mL) and high-level cross-resistance to other rifamycins. The prevalence of a specific clonal strain in the regions could explain the predominance of certain patterns observed in Panama.

It is important to note that classifying resistance based only on the absence of the WT has its limitations, as it may include technical hybridization obstacles or the presence of mutations not specifically targeted by the MUT probes of the assay, resulting in indirect forms of the assay detection (2, 28).

The study identified the *rpoβ* gene as having the highest number of strains with inferred resistance. While one would expect these mutations to be detected in the WT where their corresponding MUT is absent, around 35% of these mutations were detected in the WT where the assay includes one or more MUT (Table 4). For example, WT7 corresponds to codons 526–529 and includes H526Y/H526B mutations, WT8 at codons 530–533 includes the S531L mutation, and WT3/WT4 at codons 513–519 includes the D516V mutation.

**TABLE 4:** Codons in the *rpoβ* gene where no MUT band was detected with the GenoType MTBDRplus assay

Wild-type probe	*rpoβ* codon	Mutations not detected (*n* = 55)
ΔWT2/WT3	510–517	14
ΔWT 7	526–529	13
ΔWT6/8	522–525/530–533	5
ΔWT7/8	526–533	5
ΔWT8	530–533	4
ΔWT2	510–513	3
ΔWT2/3	513–517	3
ΔWT4/5/6	522–525	3
ΔWT6/8	522–533	3
ΔWT3/4	513–519	2

For the majority of isolates with inferred resistance, a phenotypic sensitivity test was performed, confirming resistance in 63.8% of all resistant cases. The study demonstrates a good correlation between genotypic and phenotypic assays, but it also highlights the need to consider other resistance mechanisms or even the presence of heteroresistance, thus needing both genotypic and phenotypic assays as complementary approaches for accurate resistance diagnosis (29–31).

Mutations in the *katG* gene have frequently been associated with mutations in the *rpoβ* gene, rendering this mutation a robust predictor of MDR strains (32). Furthermore, they have exhibited relative frequencies concerning mutations in INH-resistant isolates, displaying geographical variations across distinct regions (33). The *katG* S315T mutation emerged as the most prevalent, signifying high-level drug resistance, observed in up to 94% of INH-resistant isolates and most commonly in MDR strains (34).

Approximately 11.1% (42/377) of the strains exhibited low-level resistance, with the inhA C-15T mutation being the most prevalent within this subgroup, consistent with findings from prior investigations (25, 34–36). This mutation supports the utilization of high isoniazid dosages for patient treatment. These observations align with studies conducted in the South American region, as well as a previous study in Panama, where this mutation stood as the most frequently observed alteration associated with INH resistance (14, 22, 37).

Regarding mutations within the *inhA* gene that confer high-level cross-resistance to ethionamide, a second-line pharmaceutical employed in select regimens for extensively drug-resistant or multidrug-resistant patients, the incorporation of this agent into the therapeutic regimen does not yield discernible benefits. Thus, alternative therapeutic approaches warrant consideration in such cases.

In the strains where dual mutations were detected, the most frequent mutations were the S531L in *rpoβ* and the S315T1 mutation in *katG*. These findings are in concordance with results derived from a study conducted in Georgia, where these same mutations exhibited a higher prevalence among multidrug-resistant patients, considering the low fitness associated with *rpoβ* S531L mutation, contributing to its increased frequency (38).

The clinical information of the patient stands as a pivotal determinant to improve disease control, synergistically with genetic variation insights from the strains. This combination enables the correlation of alterations in transmission capability and the ability to acquire drug resistance (28). It is essential for tailoring effective treatment regimens to enhance patient outcomes and curb the spread of resistant strains. Understanding common gene mutations is essential for the development of new treatments and molecular diagnostic tools. It is particularly important to identify silent or uncommon mutations that may not be detected by currently used commercial kits, as these can lead to underdiagnosis of resistance.

Detecting such mutations also supports the justification for securing funding to implement more advanced and comprehensive diagnostic tools in our routine practice. In future investigations, it is imperative to include clinical evaluation of patient samples, from which resistant strains were derived, including data such as treatment background, therapeutic regimens employed, coexisting conditions (e.g., diabetes, immunosuppressive disorders, human immunodeficiency virus, among others), and associated risk factors (drug abuse, incarcerated individuals, healthcare personnel, among others). These data would facilitate the pursuit of diverse analyses, seeking associations that can exhibit valuable insights for the management of tuberculosis patients.
